# Unraveling Nutritional Regulation of Tacrolimus Biosynthesis in *Streptomyces tsukubaensis* through *omic* Approaches

**DOI:** 10.3390/antibiotics7020039

**Published:** 2018-05-01

**Authors:** María Ordóñez-Robles, Fernando Santos-Beneit, Juan F. Martín

**Affiliations:** 1 Área de Microbiología, Departamento de Biología Molecular, Universidad de León, León 24071, Spain; mordr@unileon.es; 2 Instituto de Biotecnología de León, INBIOTEC, Avda. Real no. 1, León 24006, Spain; fersanben3@yahoo.es; 3 Departamento de Biología Funcional, Universidad de Oviedo, Oviedo 33006, Spain

**Keywords:** *Streptomyces tsukubaensis*, tacrolimus, FK506, *omics*

## Abstract

*Streptomyces tsukubaensis* stands out among actinomycetes by its ability to produce the immunosuppressant tacrolimus. Discovered about 30 years ago, this macrolide is widely used as immunosuppressant in current clinics. Other potential applications for the treatment of cancer and as neuroprotective agent have been proposed in the last years. In this review we introduce the discovery of *S. tsukubaensis* and tacrolimus, its biosynthetic pathway and gene cluster (*fkb*) regulation. We have focused this work on the *omic* studies performed in this species in order to understand tacrolimus production. Transcriptomics, proteomics and metabolomics have improved our knowledge about the *fkb* transcriptional regulation and have given important clues about nutritional regulation of tacrolimus production that can be applied to improve production yields. Finally, we address some points of *S. tsukubaensis* biology that deserve more attention.

## 1. Discovery of *S. tsukubaensis* and Tacrolimus Use in Current Clinics

*Streptomyces tsukubaensis* and its secondary metabolite tacrolimus were discovered in 1984, during a screening performed by the Fujisawa Pharmaceutical Co. (since 2005 merged to Yamanouchi Pharmaceutical Co. to form Astellas Pharma). *S. tsukubaensis* was isolated from a soil sample in the Tsukuba region (Japan) and tacrolimus was identified in its culture broths, becoming the first immunosuppressant discovered with macrolide structure [1,2]. The strain, patented as *S. tsukubaensis* No. 9993, is currently known as *S. tsukubaensis* NRRL 18488 and is the parental strain of most of the strains used for the industrial production of tacrolimus. 

**Figure 1:**
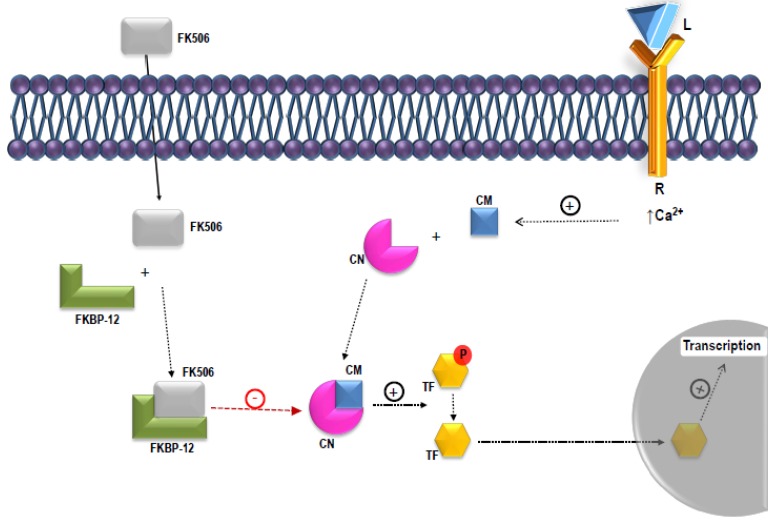
Mechanism of action of tacrolimus (FK506). Tacrolimus interacts with cytosolic receptors such as FKBP12. The complex FKBP12-FK506 inhibits the calmodulin-dependent serine/threonine phosphatase activity of calcineurin. In this situation, calcineurin can no longer dephosphorylate transcriptional factors (e.g., NFAT). The dephosphorylated TFs are required for governing T cell proliferation. L: ligand; R: receptor; CM: calmodulin; CN: calcineurin; TF: transcription factor; P: phosphate group; FKBP-12: FK506 binding protein 12.

Macrolides such as erythromycin are composed of 14–16 C-membered macrolactone rings to which one or more deoxysugars are attached. Tacrolimus, a 23-carbon macrolide (822 Da), was initially named as compound FR900506 but, later on, it received other names such as FK506 or fujimycin. The name of tacrolimus was established as an acronym of “Tsukuba Macrolide Immunosuppressant” [3]. The first reference to tacrolimus was made at the 11th International Congress of the Transplantation Society, held in Helsinki in 1986, one year before the first publications by Kino and coworkers. The first clinical assays, focused on hepatic transplantation, were developed at the University of Pittsburgh in 1989. Two years later the first international congress on tacrolimus was celebrated in that city [3]. Tacrolimus acts as a calcineurin inhibitor, showing a mechanism of action very similar to that of cyclosporine (Figure 1) [4]. When tacrolimus interacts with its cytosolic receptors, mainly FKBP12- [5], the calmodulin-dependent serine/threonine phosphatase activity of calcineurin is inhibited, resulting in the arrest of T cell proliferation [6]. The mechanism of action is conserved in human T cells and yeast and thus, tacrolimus also has antifungal activity [7,8]. This activity is useful for the qualitative detection of tacrolimus by bioassay against susceptible strains such as *Saccharomyces cerevisiae* TB23 [9].

Since its approval by the FDA for the treatment of hepatic transplantation in 1994, tacrolimus has been also applied to medulla, kidney and heart transplantation [10,11,12]. This macrolide is also used for the treatment of other diseases such as atopic dermatitis [13,14] and is applied to the stents implanted in coronary arteries [15]. Several works have been published about its use in immune diseases such as rheumatoid arthritis and intestinal inflammatory diseases [16,17]. Tacrolimus has shown antiviral activity against orthopoxvirus, HIV and feline immunodeficiency virus (FIV) [18,19,20,21] and has properties such as a hair growth stimulator [22]. Neuroprotective and neuroregenerative activities have been also reported [23,24,25] as well as its potential application in the treatment of cancer [26]. More recently, the efficacy of tacrolimus ointment in the treatment of allergic ocular diseases has been reported [27].

The efficacy of tacrolimus in the treatment of organ transplantation is the basis of its industrial importance. Tacrolimus is between 10 and 100 times more potent than cyclosporine and has been shown to be more effective in several clinical trials [28,29]. Tacrolimus generates important benefits for the pharmaceutical market; for example, the sales of tacrolimus under the commercial names ‘Prograf’ and “Protopic” yielded a total of $1727 million to Astellas Pharma in 2016 (data from http://www.pharmacompass.com).

## 2. Biosynthetic Pathway and Gene Cluster

**Figure 2:**
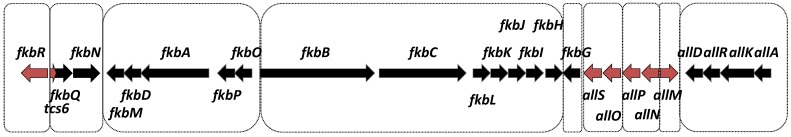
Tacrolimus biosynthesis cluster (*fkb*). Genes present in both the short and extended version of the *fkb* cluster are depicted in black. Genes present only in the extended version are depicted in red. These groups also correspond to their FkbN transcriptional dependence (**Black**) or independence (**Red**). The transcriptional units identified to date are indicated by boxes.

The first studies on the tacrolimus biosynthetic pathway were performed by researchers from the pharmaceutical company Merck (USA) during the 90’s [30,31,32,33]. Tacrolimus is a polyketide synthesized by a hybrid polyketide I synthase-non-ribosomal peptide synthase (PKSI-NRPS) system encoded by the *fkb* cluster, which encompasses a minimum of 19 genes (Figure 2). Until now, more than 15 tacrolimus-producing species have been reported [34], the last being *S. tsukubaensis* F601 [35]. There are two types of *fkb* clusters in the tacrolimus producing strains [36]: (i) A short version comprising the genes *fkbQ*, *fkbN*, *fkbM*, *fkbD*, *fkbA*, *fkbP*, *fkbO*, *fkbB*, *fkbC*, *fkbL*, *fkbK*, *fkbJ*, *fkbI*, *fkbH*, *fkbG*, *allD*, *allR*, *allK* and *allA* (found in *Streptomyces tacrolimicus* and *Streptomyces kanamyceticus* KCTC 9225) and; (ii) An extended version found in *S. tsukubaensis* NRRL 18488, *S. tsukubaensis* L19 and *Streptomyces* sp. KCTC 11604BP that includes 5 additional genes in the 5’ region of the *fkbG gene* (*allMNPOS*/*tcs12345*) and one or two extra genes (depending on the species) in the 3’ region (*tcs6*-fkbR/*tcs67*). Deletion of *allMNPOS* genes in *Streptomyces* sp. KCTC 11604BP does not significantly affect tacrolimus production; thus, it is dubious that they are involved in tacrolimus biosynthesis [37]. Actually, their transcription levels are low, which supports this assumption [38,39].

**Figure 3:**
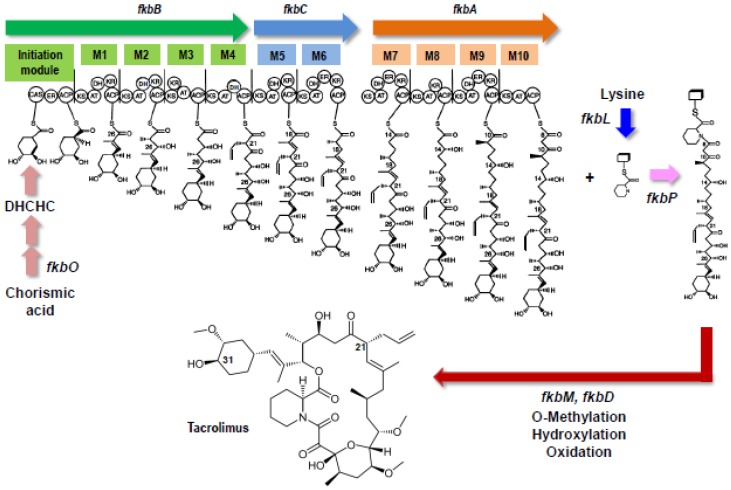
Scheme representing the assembly of the tacrolimus polyketide and the early and late biosynthetic steps. In the upper part the arrows represent the three PKS genes (*fkbA*, *fkbB*, *fkbC*) of the cluster. Note that the *fkbA* gene is physically separated from *fkbB* and *fkbC* genes in the *fkb* cluster (see Figure 2). The modules of the PKSs are boxed and indicated as M1 to M10. Domains in the modules are indicated by circles: ACP, acyl carrier protein; AT, acyltransferase; ER, enoyl reductase; CAS, CoA synthetase; KR, 3-oxoacyl (ACP) reductase; DH, 3-oxoacyl thioester dehydratase; KS, 3-oxoacyl (ACP) synthase. DHCHC: (4R, 5R)-4,5-dihydroxycyclohex-1-enecarboxylic acid. Biosynthetic and late modification steps, and the encoding genes for the starter (*fkbO*), elongation units (*fkbL*, *fkbP*) and late modification reactions (*fkbM*, *fkbD*). Based on data from Motamedi and Shafiee [30].

The first step in tacrolimus biosynthesis is the formation of (4R, 5R)-4,5-dihydroxycyclohex-1-enecarboxylic acid (abbreviated DHCHC) from chorismate through the so-called chorismatase activity of FkbO (Figure 3) [40]. DHCHC acts as starter unit for the subsequent formation of the carbon skeleton and corresponds to the cyclohexane ring in the final structure of tacrolimus. This ring is the most tolerant target for structural modifications that do not eliminate the immunosuppressant activity [41]. The polyketide synthases FkbA, FkbB and FkbC catalyze 10 elongation steps from DHCHC using as extender units malonyl-CoA (2 molecules), methylmalonyl-CoA (5 molecules), methoxymalonyl-ACP (2 molecules) and allylmalonyl-CoA (one molecule). The latter two extender units are unusual in the formation of polyketides and result in the methoxyl group of C13 and C15 and the allyl radical of C21, respectively [36,37,42,43]. The biosynthesis of methoxymalonyl-ACP from 1,3-biphosphoglycerate depends on the enzymes encoded by the *fkbGHIJK* subcluster [42,43,44,45]. The incorporation of allylmalonyl-CoA is the sole difference between tacrolimus and ascomycin (FK520), in which biosynthesis ethylmalonyl-CoA is used instead. The *all* subcluster is involved in the formation of allylmalonyl-CoA and encodes a polyketide synthase of unusual structure [46]. Nevertheless, ketoreductase and dehydratase activities encoded outside the *fkb* cluster might be involved in some steps of allylmalonyl-CoA formation and these activities could be shared with fatty acid synthases [37]. The tacrolimus cluster does not encode an ACP-CoA transacylase necessary for the final reaction leading to allylmalonyl-CoA [37], but the acyltransferase domain of the fourth module in FkbB (AT4FkbB) is able to transfer an allylmalonyl unit to the ACP domain [47].

For the cyclation of the macrolide, FkbL generates l-pipecolate from l-lysine [48], which is then incorporated into the carbon skeleton by NRPS FkbP [30,49,50]. Finally, two modification steps are necessary to achieve the final molecule with biological activity: a methylation of the hydroxyl group located at C31 and an oxidation at C9. Both groups are important for the binding of tacrolimus to FKBP12 [51,52]. The methylation is catalyzed by the S-adenosylmethione dependent O-methyltransferase FkbM and the oxidation by the cytochrome P450-oxidoreductase FkbD [31,53]. Both activities are encoded in the same operon and can occur in any order [31,45,54]. Interestingly, the reaction catalyzed by FkbD (a double step oxidation involving 4 electron transfers and the formation of the alcoholic intermediate 9-hydroxy-FK506) is known for terpenoid biosynthesis but was first described for polyketide biosynthesis [45].

## 3. Transcriptional Regulators and Recent Insights through Transcriptomic and RNAseq Studies

The first sequence analyses of the *fkb* cluster revealed three potential regulators: *fkbN*, *fkbR* and *allN* (belonging to the LAL, LysR and AsnC families, respectively). FkbN is a large regulatory protein of the LAL family (Large ATP binding regulators of the LuxR family). The LAL regulators are large proteins (872–1159 amino acids) that contain a LuxR-type HTH DNA binding region near the C-terminal end of the protein and an ATP binding motif in the N-terminal end [55,56]. Similar FkbN-like genes have been found in several other macrolide gene clusters including RapH of the rapamycin producer *Streptomyces hygroscopicus* [57], PikD of the pikromycin producer *Streptomyces venezuelae* [58], GdmR1 and GdmR2 of the geldanamycin producer *Streptomyces hygroscopicus* [59], FkbN of the ascomycin producer *S. hygroscopicus* var. *ascomyceticus* [44], FscRI in the candicidin producer *Streptomyces griseus* [60,61], PimM of the pimaricin producer *Streptomyces natalensis* [62,63], NysR from the nystatin producer *Streptomyces noursei* [64], AmphRIV in the amphotericin B producer *Streptomyces nodosus* [65] and PteF in the filipin producer *Streptomyces avermitilis* [66,67].

The second regulatory protein FkbR belongs to the family of the LysR-type transcriptional regulators, also named LTTR, which are very common autoregulatory genes in bacteria [68]. In fact, they are widely distributed in *Streptomyces*: genome sequencing revealed about 40 LTTRs in *S. coelicolor* [69]. FkbR, as occurs with other members of the LTTR family, is a relatively small protein of less than 325 amino acids that is characterized by an HTH DNA binding motif in the C-terminal and by a ligand (co-inducer) binding sequence in the N-terminal region [70,71]. Other LTTRs acting as pathway-specific regulators include SCLAV_p1262 of *S. clavuligerus* (77% identity), ThnI from *Streptomyces cattleya* (39% identity), AbaB from *Streptomyces antibioticus* or ClaR from *S. avermitilis* [72,73].

The third putative regulatory gene of the tacrolimus gene cluster is *allN*. This gene is located in the 5’ end of the extended version of the tacrolimus gene cluster and encodes a protein that has similarity with regulatory proteins involved in nitrogen metabolism, particularly with regulators of AsnC family [74]. This gene is included in a region that is involved in the formation of the precursor allylmalonyl-CoA (all genes) [37,46].

Functional analysis of the role of FkbN, FkbR and AllN in *S. tsukubaensis* was performed by gene disruption and complementation studies. Whilst the inactivation of *fkbN* resulted in the lack of tacrolimus production, disruption of *fkbR* reduced tacrolimus yields to 20% of that of the parental strain and the inactivation of *allN* did not affect tacrolimus production [36]. Thus, it was concluded that both *fkbN* and *fkbR* encode positive regulators whilst *allN* has no influence on tacrolimus production [36]. In addition, AllN (also named Tcs2) seems to be not involved in tacrolimus production in other strains such as *S. tsukubaensis* L19 [75]. Overexpression of *fkbN* or *fkbR* in the wild type strain using the ermE* promoter produced an increase of the final yield of tacrolimus of 55% and 30%, respectively, using a culture medium optimized for tacrolimus production. These results agree with the observations published by Mo and coworkers on the effect of FkbN in *Streptomyces* sp. KCTC 11604BP [76].

**Figure 4:**
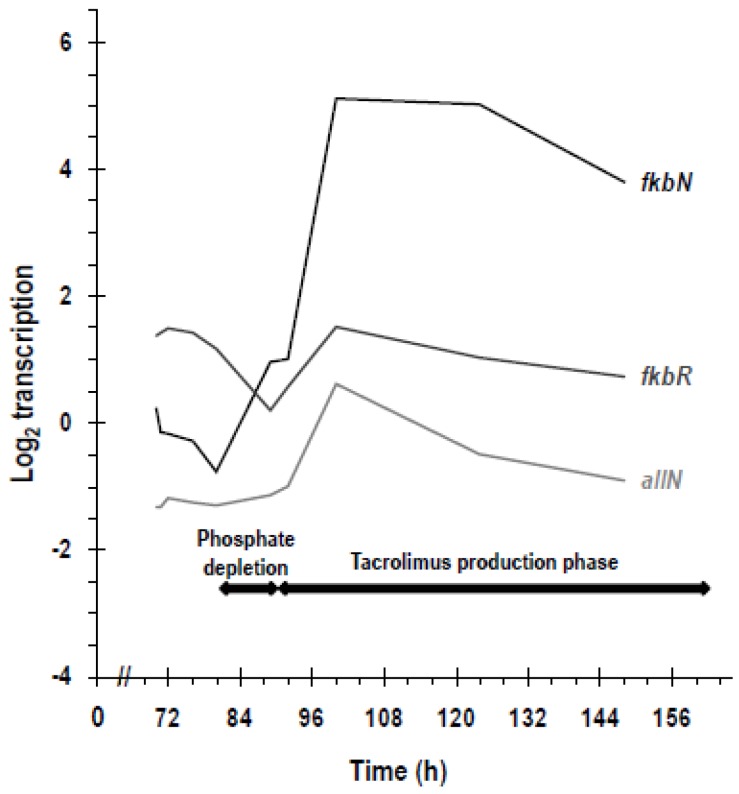
Transcriptional profiles of genes encoding transcriptional regulators of the *fkb* cluster. Transcription of *fkbN*, *fkbR* and *allN* in *S. tsukubaensis* NRRL 18488 grown in MGm-2.5 production media. As indicated in the graph, phosphate depletion occurs between 80 h and 89 h and tacrolimus is detected from 89 h. The cultures were performed in duplicated flasks. Error bars have been omitted to facilitate the visualization of the results.

There are important differences between FkbN and FkbR that we summarize here as follows: (1) *fkbN* is present in both the extended and the short version of the *fkb* cluster but *fkbR* is only present in the extended cluster version [37]; (2) FkbN always shows a positive effect on tacrolimus production whilst FkbR can have positive or negative effects [36,76,77]; (3) A complete lack of tacrolimus production is only produced by inactivation of *fkbN* (but not with that of *fkbR*) [36,38]; (4) transcription of *fkbR* is constant and low throughout the culture whilst that of *fkbN* increases before the onset of tacrolimus production and is maintained during the production phase (Figure 4) [36,38,75].

### 3.1. Characterization of fkb Cluster Transcriptional Subunits

Early studies using the *rppA* chalcone synthase reporter systems and qRT-PCR showed that the inactivation of *fkbR* or *fkbN* prevents transcription of certain genes in the *S. tsukubaensis fkb* cluster such as *fkbG* or *fkbB*, implying that some *fkb* genes are regulated by FkbN while others are not [36]. However, more recent transcriptomic studies with the same *fkbN* inactivated mutant have confirmed that FkbN controls the expression of most of the genes of the *fkb* cluster [38]. Two types of gene expression were observed in response to *fkbN* inactivation: (a) Genes clearly induced by FkbN coinciding with the onset of tacrolimus biosynthesis (in the so called “induction phase”) and whose expression is significantly reduced in the *fkbN* mutant (i.e., *fkbABC*, *fkbGHIJK*, *fkbL*, *allAKRD*, *fkbO*, *fkbP*, *fkbD* and *fkbM*) and (b) Genes poorly expressed through the culture time and not affected by *fkbN* inactivation (i.e., *allMNPOS* and *fkbR*) (Figure 2). Thus, the complete transcriptional dependency of the *fkb* genes on FkbN, with the exception of *allMNPOS* and *fkbR* (only present in the extended versions of the *fkb* cluster), which are FkbN-independent, was demonstrated.

The use of tiling probes covering the *fkb* cluster allowed the identification of 6 transcriptional units: *fkbR*, *tcs6-fkbQ-fkbN*, *fkbOPADM*, *fkbBCLKJIH*, *fkbG* and *allAKRD*. It was concluded that *fkbR* is transcribed as a leaderless mRNA and that *fkbN* forms an operon along with *tcs6* and *fkbQ* whose transcription depends on two different promoters, one FkbN-dependent and the other FkbN-independent [38]. These results are supported by the EMSAs performed with the FkbN-DNA binding domain in *S. tsukubaensis* L19 by Zhang and coworkers [75], who reported FkbN binding to the promoter regions of the same six transcriptional units and identified two new ones corresponding to *allNPOS* and *allM*. More recently, differential RNA-seq (dRNA-seq) transcriptional profiling has been performed in *S. tsukubaensis* by Bauer and coworkers [39], who identified 9 transcriptional units that are in good agreement with previous studies (Figure 2). The main finding is that *allOS* and *allNP* are transcribed as independent mRNAs [39].

*fkbR* seems to be transcribed as a leaderless mRNA and is not directly regulated by FkbN [38]. In fact, it is likely that FkbR regulates its own expression, although detailed information is not available. Recently, the binding of FkbR to the promoter regions of *tcs6-fkbQ-fkbN* and *fkbR* in *S. tsukubaensis* L19 has been reported [75].

### 3.2. Genes Located Outside of the Tacrolimus Gene Cluster Regulated by FkbN

It has been reported that cluster-situated regulators (CSR) can regulate genes located outside their own cluster [78,79] and, therefore, the utilization of transcriptomic studies is a good tool to identify them. The transcriptomic analysis performed with the *fkbN* mutant by Ordóñez-Robles and coworkers [38] revealed potential genes located outside the *fkb* cluster that might be targets of FkbN such as *ppt1*, encoding a 4′-phosphopantetheinyl transferase that is known to be involved in CDA formation in *S. coelicolor* [80]. This gene showed an FkbN-dependent profile and a putative FkbN binding sequence [38]. In agreement with these results it was reported that the orthologue of *ppt1* is involved in tacrolimus production in *S. tsukubaensis* L19 [81] and later, it was observed that *ppt1* and *fkbN* share a common transcriptional response to glucose, glycerol and *N*-acetylglucosamine additions (see below). The study identified acyl-CoA dehydrogenase and methoxymalonate biosynthesis coding genes that were negatively affected by the *fkbN* inactivation and thus, might be involved in tacrolimus biosynthesis. On the contrary, some PKS coding genes located in a chromosomal region that has been predicted to encode a cluster for the production of a bafilomycin-like compound [82] were upregulated after *fkbN* inactivation, which might reflect competition for precursors between these two clusters for the biosynthesis of secondary metabolites.

Using the information-theory of Schneider [83], a putative FkbN binding sequence would be composed by two 7 nt inverted repeats [38]. This sequence would be similar to that identified for binding of PimM in the genome of *S. natalensis* [63].

In-depth knowledge of the *fkb* cluster regulation is necessary to achieve higher tacrolimus production yields. In this sense, the identification of transcriptional start sites (TSS) is useful for the introduction of artificial promoters without affecting the structure of mRNAs. Bauer and coworkers [39] reported that 22% of the transcripts identified by dRNAseq are predicted to present long leader mRNAs (greater than 150 nt), which points out the importance of post-transcriptional regulation of the *fkb* cluster through the formation of RNA secondary structures [84]. In fact, the *allAKRD* operon was reported to be transcribed with a rather long untranslated 5’ region (5’-UTR; 247 bp) that is predicted to form a secondary structure.

## 4. Classical Strategies to Increase Tacrolimus Production

Despite the efficacy of tacrolimus in the treatment of organ transplantation, its use in clinical therapy is expensive. This is mainly due to the low production yields of the producer strains used but also to the formation of byproducts such as ascomycin (FK520) or FK525, which are structurally similar to tacrolimus but differ in the nature of some radical groups [85]. The presence of byproducts in the culture broths hampers extraction and purification of tacrolimus; thus, different approaches involving the use of organic solvents and/or chromatography have been developed to increase tacrolimus purity [86]. As an example, ascomycin production can represent 20% of tacrolimus production in *S. tsukubaensis* NRRL 18488 and 8% in *Streptomyces clavuligerus* KCTC 10561BP [86,87]. The chemical synthesis of tacrolimus was described in the 90’s but it is not applied in practice due to its low efficacy and high costs [88,89].

In the last decades, the research on tacrolimus production enhancement has been mainly focused on culture media optimization and genetic engineering of the strains. For a recent review on the improvement of tacrolimus biosynthesis through synthetic biology approaches see [90,91]. The optimization of culture media encompasses formulation of defined compositions, precursor supply and the addition of stressing agents. Defined media are highly necessary to perform nutritional studies in which the stimulating or inhibitory effect of a particular nutrient on growth and antibiotic production is tested. The first defined media for the growth of *Streptomyces* sp. MA6858 (ATCC 55098) was formulated by Yoon and Choi [92]; later, Martínez-Castro and coworkers [93] developed two additional media, MGm-2.5 and ISPz. MGm-2.5, which contain starch as the main carbon source and glutamate as carbon and nitrogen sources whilst ISPz, an optimization of ISP4 medium, contains glucose and corn dextrin as the main carbon source. MGm-2.5 has been further used to perform transcriptomic analyses on the carbon and phosphate control of *S. tsukubaensis* [94,95]. This medium supports dispersed growth and high tacrolimus production yields. Moreover, this medium permits an estimate of the onset of tacrolimus production since this process has been shown to take place when phosphate is depleted from this medium [93].

Considering that the availability of precursors is a limiting factor in the biosynthesis of secondary metabolites, precursor supply is a straightforward strategy to increase antibiotic yields [96]. A summary of the compounds that have been applied to increase tacrolimus production is shown in Table 1. At this point of the review and as a conclusion of all the mentioned work, it is interesting to note that (1) The effect of a precursor depends on its concentration; (2) The combination of positive additions does not always have an additive positive effect and (3) The positive effect can be exerted through growth promotion, production stimulation or both.

Nevertheless, the addition of precursors in industrial fermentations can be a non-efficient strategy from an economical point of view (i.e., shikimate, chorismate and pipecolate are expensive; [107]); thus, an alternative strategy is to increase the copy number of tacrolimus biosynthetic genes by genetic engineering. In this manner, the overexpression of genes coding for the synthesis of methylmalonyl-CoA, methoxymalonyl-ACP and allylmalonyl-CoA has been shown to have a positive impact on the tacrolimus production yields [104,108].

Finally, the addition of stressing agents, such as dimethylsulfoxide (DMSO) or sodium thiosulfate, has been shown to stimulate polyketide production in different bacteria [109,110] as well as tacrolimus production in *S. tsukubaensis* NRRL 18488 [90].

## 5. Omic Approaches in *S. Tsukubaensis* and Their Application in Tacrolimus Production

### 5.1. Metabolomic and Proteomic Studies

The inactivation or overexpression of a particular gene involved in a certain biosynthetic pathway can affect other metabolic pathways and also the growth of the microorganism. For this reason, global studies covering the whole transcriptome, proteome or metabolome are usually preferred. In *S. tsukubaensis*, several metabolomic studies have been performed in the last decade. Huang and coworkers [100,111] developed a genome-scale metabolic model (GSMM) for *S. tsukubaensis* D852 including 865 chemical reactions and 621 metabolites to predict targets for genetic manipulation. These models reconstruct the organism metabolism from the genome annotation, taking into account genes encoding enzymes and transporters. By this means it was predicted that some of those modifications in the primary metabolism pathways leading to the accumulation of erythrose-4-phosphate, α-ketoglutarate, fumarate, succinate, pyruvate, phosphoenolpyruvate, NADPH, chorismate and malonyl-CoA have a positive effect on tacrolimus production. This implies that both the pentose phosphate pathway and the TCA cycle are positively correlated with tacrolimus production. Regarding the biosynthetic cluster, the overexpression of genes involved in the formation of the starter unit DHCHC, pipecolate and in different modification reactions (*fkbO*, *fkbL*, *fkbP*, *fkbM* and *fkbD*; see Table 2) also has a positive effect. Interestingly, as mentioned before, the combination of positive mutations does not always have an additive effect, i.e., the combined overexpression of *fkbL* and *fkbP* reduced biomass formation due to the use of lysine for tacrolimus production. More recently, a metabolomic approach has been reported in which lysine, shikimate, malonate, and citrate (the last three ones in the form of sodium salts) were supplied to the culture media of *S. tsukubaensis* D852 [102]. In this study, the addition of compounds targeting different precursor pathways facilitates the comprehension of the metabolic switches that are positive for tacrolimus production, and the application of weighted correlation network analysis (WGCNA; [112]) allowed the identification of hub modules and key metabolites depending on the culture stage. For example, 48 h after the feeding, pyruvate, phosphoenolpyruvate and methylmalonate show a high degree of connectivity whilst 72 h after the feeding, shikimate and aspartate control tacrolimus production. Supporting previous results, it was reported that the pentose phosphate, shikimate and aspartate pathways are crucial for the biosynthesis of the immunosuppressant. Overexpression of *aroC* and *dapA* (involved in shikimate pathway and lysine biosynthesis, respectively) increased production of the macrolide by 40% and 23%, respectively. See a summary of the distinct gene modifications that produce a positive impact on tacrolimus production in Table 2.

The GSMM developed by Huang and coworkers [111] is a pseudo-steady metabolic model, that is to say, it assumes that there is no depletion or accumulation of intracellular metabolites. Dynamic flux balance analysis (DFBA) takes into consideration the fluctuations in metabolite concentrations and thus allows the study of the interaction between metabolism and environmental changes [114]. Wang C. and coworkers [113] developed a genome-scale DFBA (GS-DFBA) model for *S. tsukubaensis* NRRL 18488 which uncovered new targets for genetic manipulation (see Table 2) that resulted in increased tacrolimus production; i.e., inactivation of *gcdh* (glutaryl-CoA dehydrogenase) and overexpression of *tktB* (transketolase), *msdh* (methylmalonate semialdehyde dehydrogenase) and *ask* (aspartate kinase).

The approached used by Xia and coworkers [99] consisted of the growth of *S. tsukubaensis* TJ-04 in two media of similar composition but resulting in different tacrolimus productivity. They analyzed the concentration of a wide range of metabolites and compared them between the two media to identify key metabolites that correlate positively with tacrolimus production. In good agreement with the results of Huang and coworkers [100,111], intermediates of the TCA cycle such as oxaloacetate, citrate, α-ketoglutarate and, especially, succinyl-CoA and acetyl-CoA, showed a positive correlation with tacrolimus production. In addition, the intracellular levels of pentose phosphate pathway intermediates were lower in the high production media, supporting the assumption that this pathway is positively correlated with tacrolimus production. Regarding metabolites from the tacrolimus biosynthetic pathway, methylmalonyl-CoA showed the best correlation.

More recently, Wang and coworkers [103] performed a comparative proteomic and metabolomic approach in *S. tsukubaensis* NRRL 18488 grown under soybean oil feeding. The positive effect of this carbon source on growth and on tacrolimus production has been already reported in other producing strains [97,98,99,100,101] and, as expected, increased tacrolimus production by 89%. This work has unraveled the effect of soybean oil on tacrolimus production, which mainly affects primary metabolism proteins (42%), redox proteins (12.5%), transcriptional regulators, signal transduction components and translation proteins (11%). The key metabolites associated with tacrolimus production correlate well with those identified previously by Xia and coworkers [99] and include malic acid, gluconic acid, citric acid, α-ketoglutarate, hexadecanoic acid, threonine, fumaric acid, succinic acid, proline, valine, oleic acid, trehalose, pyruvate, ornithine, 10-undecenoic acid, shikimic acid, mannose, and lactate. Several enzymes involved in the lower glycolytic pathway and the TCA cycle (i.e., triosephosphate isomerase, phosphoglycerate mutase, pyruvate kinase or citrate synthase) were overproduced under the soybean oil condition, and the rate-limiting enzyme of the pentose phosphate pathway glucose-6-phosphate dehydrogenase showed higher amounts in the fed condition, which supports the above-mentioned positive correlation of the pentose phosphate and TCA cycle pathways with the tacrolimus production process. Finally, enzymes related to fatty acid, shikimic acid, valine and isoleucine metabolisms (which can be transformed in the extender units methylmalonyl-CoA and propionyl-CoA) were also upregulated (valine and isoleucine can be transformed in the extender units methylmalonyl-CoA and propionyl-CoA). Interestingly, higher amounts of the transcriptional regulators Crp and AfsQ1 were detected under the soybean oil feeding condition, pointing to their possible involvement in tacrolimus production regulation.

### 5.2. Transcriptomic Studies on Phosphate Regulation of the fkb Cluster

Understanding how a biosynthetic cluster is regulated is important to develop strategies to improve secondary metabolite production. Our group has studied the phosphate regulation of antibiotic production in different *Streptomyces* species in the last two decades, including *S. tsukubaensis* [94,115,116]. It is well known that high phosphate concentrations in the culture media downregulate antibiotic production [117]. This regulatory phenomenon is exerted, at least in part, through the two-component system PhoR-PhoP, which is formed by a sensor kinase and a response regulator, respectively [115,118]. When phosphate is depleted from the culture media, PhoR phosphorylates PhoP. The binding of phosphorylated PhoP (PhoP-P) to its target sequences (known as PHO boxes) can have a positive or negative transcriptional effect depending on the location of the PhoP-P binding site [118,119,120]. In *S. tsukubaensis*, the negative regulation of tacrolimus biosynthesis by phosphate was reported in 2013 [93] and later the PhoR-PhoP system was studied in detail [94]. In the work, transcriptomics were applied to identify genes that are transcriptionally activated after phosphate depletion. The study allowed the identification of not only common Pho members but also of potential new species-specific members, like, for example, three overlapping genes encoding a two component system and a small hydrophilic protein. In addition, a bioinformatic search for PHO boxes was developed [121]. Putative PHO boxes were identified in most of the genes responding to phosphate starvation, supporting the transcriptional results. A putative PHO box was identified in the promoter region of *fkbN* and also in primary metabolism genes that might be involved in tacrolimus precursor supply such as STSU_30046, encoding an acetoacetate-CoA ligase [94].

#### 5.2.1. Transcriptomics of Carbon Catabolite Regulation of Tacrolimus Biosynthesis

A second regulatory mechanism governing secondary metabolite production is carbon repression. Similar to phosphate, the presence of ready-to-use carbon sources in the media reduces or blocks antibiotic production and this can happen at the transcriptional or at the posttranslational level [122,123]. The mechanisms involved in this nutritional regulation are not completely understood in streptomycetes and, as it can be deduced, its unveiling is very interesting in order to use easily assimilated carbon sources that allow faster growth in the culture broths without hampering tacrolimus biosynthesis. Regarding this subject, our group observed that glucose and glycerol, when added as carbon sources at a concentration of 0.22 M at the first growth phase (and before phosphate depletion), arrest tacrolimus production in *S. tsukubaensis*; the glucose effect being stronger than that of glycerol [95]. Both glucose and glycerol additions resulted in a lack of transcriptional activation of the *fkb* cluster; thus, it was concluded that transcriptional repression plays a role in this regulatory mechanism. In addition, the effect of these carbon sources can be exerted at the intermediary metabolism level: glucose addition increased transcription of genes involved in glycolysis, pyruvate and oxaloacetate formation but downregulated genes involved in the TCA cycle. These results are coherent with the previous assumption that the TCA cycle is positively correlated with tacrolimus production whilst glycolytic metabolites show a negative correlation [99].

In the MGm-2.5 medium used in the work, transcription of *fkbN* increases in a two-step fashion before tacrolimus is detected in the broths [38]: a slight increase in mRNA levels occurs between 80 h and 89 h and then it is followed by a higher increase from 92 h to 100 h (Figure 4). The first step coincides with phosphate depletion, supporting the proposal that *fkbN* is under phosphate control [95] (Figure 4). Taking into account that *fkbN* transcription is not strongly self-regulated [38], it seems that a key transcriptional regulator, co-activator molecule or sigma factor might be absent in the presence of glucose or glycerol. Therefore, the identification of this additional factor would be useful to trigger tacrolimus production under carbon repressing conditions. Actually, key sigma factors (i.e., *hrdA* or *bldN*) and transcriptional regulators (i.e., *eshA*, *atrA*, *afsR*) were downregulated under glucose or glycerol addition conditions [95]. HrdA might control secondary metabolism genes [124], and EshA and AtrA are both involved in antibiotic production in *S. coelicolor* and *S. griseus* [125,126,127,128]; thus, it seems interesting to analyze the effect of their inactivation and overexpression on tacrolimus production. Finally, AfsR is a very interesting candidate for these studies since it is overexpressed in an *S. tsukubaensis* strain that overproduces tacrolimus [101].

#### 5.2.2. Transcriptomics of *N*-acetylglucosamine Addition in Tacrolimus Biosynthesis

A third example of the nutritional regulation of secondary metabolite production is that exerted by *N*-acetylglucosamine, the monomer of chitin. This compound shows a dual regulatory role, accelerating differentiation and antibiotic production under poor nutritional conditions and arresting them under rich nutritional conditions, which have been traditionally named as “famine” and “feast” conditions, respectively [129,130]. We observed a negative effect of *N*-acetylglucosamine addition on tacrolimus production when *S. tsukubaensis* was grown in MGm-2.5 medium, which might be due, at least in part, to the transcriptional repression of *fkbN*, since we observed a significant decrease in its transcription soon after *N*-acetylglucosamine addition (Ordóñez-Robles et al., unpublished data). The transcriptional response to *N*-acetylglucosamine addition is very similar to that exerted by glucose, which is not surprising since both carbon sources share a common catabolic pathway from fructose-6-phosphate.

Overall, the application of transcriptomics to nutritional studies in *S. tsukubaensis* unveils potential candidates for the rational engineering of industrial strains. It has also improved our knowledge about other aspects of its physiology such as the possible members of the PHO regulon in this species or the mechanisms operating in the presence of repressing carbon sources. These findings are worthy to detect potential targets for the bypass of nutritional repression of secondary metabolism in *Streptomyces*.

## 6. Conclusions and Future Prospective

It has been more than 30 years since *S. tsukubaensis* and its secondary metabolite tacrolimus were discovered. Despite the importance of this immunosuppressant macrolide in current clinics, there are still many aspects to be elucidated about the transcriptional and nutritional regulation of tacrolimus biosynthesis, and further studies are necessary to improve the yield and reduce the costs of its industrial production. In this sense, the *omic* approaches constitute an important basis to understand the producer microorganism physiology from a genome- [131], proteome- and metabolome-wide point of view. Initial *omic* studies performed in *S. tsukubaensis* have given important clues such as the positive correlation of the pentose phosphate pathway and TCA cycle with tacrolimus production or the identification of targets for genetic manipulation. These types of studies can be applied not only to the overproduction of tacrolimus but also to the awakening of cryptic clusters [132]. In fact, similar to most streptomycetes, *S. tsukubaensis’* genome contains several clusters for the production of secondary metabolites which might encode useful compounds. One of the potential products encoded is predicted to be similar to bafilomycin [133] and two other clusters show homology to those for biosynthesis of nigericin and enduracidin [134,135]. Nevertheless, we must keep in mind the interpretation of the *omic* results in the framework of the strain and culture media used since there are important physiological differences depending on the strain and the culture conditions. Therefore, the comparison of different models can broaden our perspective of tacrolimus production and *S. tsukubaensis’* physiology.

There are still some interesting points to address in the study of the *fkb* cluster such as the role of the *allMNPOS* subcluster in the strains that contain it. Although not strictly required for tacrolimus production, the *all* subcluster might be involved in the generation of macrolide variants with useful properties. Thus, the overexpression of these genes under promoters regulated by FkbN seems an interesting study. In addition, the *ppt1* and *scoT* genes, which are affected by the inactivation of *fkbN*, might be potential targets for tacrolimus biosynthesis improvement. Considering the transcriptional regulation of *eshA* and *atrA* under tacrolimus producing and repressing conditions, both genes seem good candidates for genetic engineering of the strains.

The transcriptional regulation of *fkbN* is also interesting given that it is the main transcriptional activator of the *fkb* cluster. The identification of transcriptional regulators that bind to its promoter region is a good approach to identify new targets for genetic engineering of the strains that overexpress *fkbN* and therefore, to increase tacrolimus production. Finally, the post-transcriptional regulation of the *fkb* cluster deserves further attention. As reported by Bauer and coworkers [39], a high percentage of genes are transcribed with long leader sequences in *S. tsukubaensis* (i.e., *allAKRD*). Long 5’-UTRs might be involved in the formation of secondary structures that regulate transcription of the cistrons and might be potential targets for manipulation.

## Figures and Tables

**Table 1:** Common precursors used for tacrolimus production enhancement in different *S. tsukubaensis* strains. The precursor, *S. tsukubaensis* strain used and bibliographic reference are indicated.

Precursor	Strain	Reference
Soybean oil	*Streptomyces* sp. MA6858 B3178	[97,98,99,100,101]
	*S. tsukubaensis* TJ-04	
	*S. tsukubaensis* D852	
l-lysine	*Streptomyces* sp. MA6858 B3178	[93,97,98,100,101,102,103]
	*S. tsukubaensis* D852	
	*S. tsukubaensis* NRRL18488	
Methyl-oleate	*S. clavuligerus* CKD1119	[98,104]
Pipecolic acid	*S. tsukubaensis* NRRL18488	[100,101,105]
	*S. tsukubaensis* D852	
Picolinic acid	*S. tsukubaensis* NRRL18488	[105]
Nicotinamide	*S. tsukubaensis* NRRL18488	[105]
Nicotinic acid	*S. tsukubaensis* NRRL18488	[105]
Chorismate	*S. tsukubaensis* D852	[100,101]
Shikimate	*S. tsukubaensis* D852	[99,100,101,102,103]
	*S. tsukubaensis* TJ-04	
	*S. tsukubaensis* NRRL18488	
Lactate	*S. tsukubaensis* D852	[92,100,101,102]
	*Streptomyces* sp. MA6858	
Succinate	*S. tsukubaensis* D852	[99,100,101,103]
	*S. tsukubaensis* TJ-04	
	*S. tsukubaensis* NRRL18488	
Isoleucine	*S. tsukubaensis* D852	[100,101]
Valine	*S. tsukubaensis* D852	[99,100,101]
	*S. tsukubaensis* TJ-04	
Proline	*S. tsukubaensis* TJ-04	[99]
Leucine	*S. tsukubaensis* TJ-04	[99]
Threonine	*S. tsukubaensis* TJ-04	[99]
Propilenglycol	*S. tsukubaensis* FERM BP-927	[106]
Propanol	*S. tsukubaensis* FERM BP-927	[106]
Propionic acid	*S. tsukubaensis* FERM BP-927	[106]
Malonate	*S. tsukubaensis* D852	[102,103]
	*S. tsukubaensis* NRRL18488	
Citrate	*S. tsukubaensis* D852	[102,103]
	*S. tsukubaensis* NRRL18488	

**Table 2:** Genetic modifications predicted through metabolic modelling in *S. tsukubaensis* to improve tacrolimus production. The target gene, type of modification, strain and bibliographic reference are indicated.

Gene/Modification	Strain	Reference
*fkbO*/overexpression	*S. tsukubaensis* D852	[100]
*fkbL*/overexpression	*S. tsukubaensis* D852	[100]
*fkbM*/overexpression	*S. tsukubaensis* D852	[100]
*fkbP*/overexpression	*S. tsukubaensis* D852	[100]
*fkbD*/overexpression	*S. tsukubaensis* D852	[100]
*gdhA*/inactivation	*S. tsukubaensis* D852	[111]
*ppc*/inactivation	*S. tsukubaensis* D852	[111]
*dahp*/overexpression	*S. tsukubaensis* D852	[111]
*pntAB*/overexpression	*S. tsukubaensis* D852	[111]
*accA2*/overexpression	*S. tsukubaensis* D852	[111]
*zwf2*/overexpression	*S. tsukubaensis* D852	[111]
*fkbD*/overexpression	*S. tsukubaensis* D852	[111]
*aroC*/overexpression	*S. tsukubaensis* D852	[102]
*dapA*/overexpression	*S. tsukubaensis* D852	[102]
*gcdh*/inactivation	*S. tsukubaensis* NRRL 18488	[113]
*tktB*/overexpression	*S. tsukubaensis* NRRL 18488	[113]
*msdh*/overexpression	*S. tsukubaensis* NRRL 18488	[113]
*ask*/overexpression	*S. tsukubaensis* NRRL 18488	[113]
